# *Infectious Diseases of Poverty*: progress achieved during the decade gone and perspectives for the future

**DOI:** 10.1186/s40249-021-00931-3

**Published:** 2022-01-04

**Authors:** Xiao-Nong Zhou

**Affiliations:** 1 National Institute of Parasitic Diseases, Chinese Center for Disease Control and Prevention; Chinese Center for Tropical Diseases Research; WHO Collaborating Centre for Tropical Diseases; National Center for International Research on Tropical Diseases, Ministry of Science and Technology; National Health Commission Key Laboratory of Parasite and Vector Biology, Shanghai, 200025 China; 2 School of Global Health, Chinese Center for Tropical Diseases Research, Shanghai Jiao Tong University School of Medicine, Shanghai, 200025 China

The journal *Infectious Diseases of Poverty* (IDP) was launched on October 25, 2012. The “One Health-One World” concept remains in focus with the least developed countries at the epicentre of the publication activities. However, IDP continues to explore new avenues in research to better understand the relationship between infectious diseases and poverty, and focuse on trans-disciplinary and multisectoral research on health systems, environmental management and innovative technology. While the coronavirus disease 2019 (COVID-19) pandemic is threatening to delay the global progress towards Universal Health Coverage (UHC), IDP has become an even more important platform assuring that scientists all over the world, be they from urban cities or remoted rural areas, not only gain access to new knowledge related to infectious diseases of poverty, but also are offered an outlet for publishing their own research [1].

The year 2022 marks the 10th anniversary of IDP. It is therefore timely to look back at the origin assessing the aims achieved, especially with respect to the developing world and the journal’s contribution to the Millennium Sustainable Goals (MDGs) and the subsequent Sustainable Development Goals (SDGs) initiated by the United Nations (UN). One of the SDGs was to identify and weigh up research and information gaps that hinder progress towards new interventions for health problems particular for the developing world. It is envisioned that IDP will successfully continue promoting global elimination of infectious diseases of poverty through trans-disciplinary research bridging the research—policy gap. Above all, IDP provides comprehensive and authoritative coverage of important topics via scoping reviews, research articles, commentaries, opinions, etc. with the aim of strongly contributing to achieving the SDGs within the “One Health” framework.

## A decade of achievements

By the end of October 2021, IDP had published a total of 940 articles selected from more than 3,927 submitted manuscripts whose authors representing 105 different countries. More than 60% of the authors are in Africa, Asia and Latin America. In an effort to support researchers with limited funding, the publication fees for nearly 30% of manuscripts from low- and middle-income countries were discounted or completely waived. Four major successful approaches were accomplished by the creation of the IDP publication platform that has advanced research and evidence building leading to improved public health interventions in poor settings.

### Elevating impact through articles on essential topics and key thematic series

Scientific issues of central interest, such as climate change, emerging infections, antimicrobial resistance, social-economic evaluation, have been continually presented with special attention paid to the Nobel Prizes awarded in the medical field. In order to attract more readers, IDP has always been looking for essential topics related to infectious diseases of poverty and we promptly publicise comments on new research findings by well-known experts in these fields. Examples include the papers on the Nobel award for the artemisinin and ivermectin discoveries in 2015, the innovative treatment of *Ascaris* infection and malaria transmission in 2016, the poverty-related infectious diseases in 2019, and the transmission patterns of COVID-19 in 2020 [2–5].

The journal’s scope covers the three most important infectious diseases (HIV/AIDS, tuberculosis, malaria), the 20 neglected tropical diseases (NTDs), and the many emerging and re-emerging infectious diseases, such as Ebola, avian influenza, and COVID-19. Essential public health questions are addressed with strong emphasis on epidemiology, modelling, control strategies, implementation of new surveillance technologies and applications, treatment and case management as shown in Table 1. In addition, IDP also coordinates the publication of 3 to 5 thematic series every year to interpret the trans-disciplinary or multi-sectoral effects on health issues.

**Table 1:** The publication record with regard to disease and number of papers published

Type of infection	Diseases	Number of articles
Viral infections	Rabies^ab^, dengue^c^	58
Bacterial infections	Tuberculosis^a^, Buruli ulcer^a^ Leprosy^a^, meningitis^a^	161
Parasitic infections		
Vector-borne diseases	Malaria^a^, Chagas disease^ac^, African trypanosomiasis^a^, onchocerciasis^ac^, lymphatic filariasis^a^, leishmaniasis^a^	209
Zoonotic diseases	Schistosomiasis^ab^, opisthorchiasis^ab^, clonorchiasis^ab^, paragonimiasis^ab^, fascioliasis^ac^, fasciolopsis^ab^, cryptosporidiosis, giardiasis, toxoplasmosis, taeniasis/cysticercosis^a^, echinococcosis^a^	149
Soil-transmitted helminthiasis	Hookworm^a^, trichuriasis^a^, ascariasis^a^, enterobiasis^a^	44
Others	NTDs, hepatitis, HIV/AIDS, COVID-19, Ebola, avian influenza	319

^a^Ancient diseases; ^b^Emerging or re-emerging infectious disease; ^c^Emerging or re-emerging infectious disease in some regions

### Dedication to the original goals in changing times

IDP was launched at a time focused on achieving the MDGs with the objective of filling the trans-disciplinary gaps in research with regard to the infectious diseases of poverty. Since 3 of the 8 MDGs were chosen to contribute to poverty alleviation and public health equity, the journal quickly found its niche. However, the scope changed to some extent in 2015 when the UN endorsed the succeeding 17 SDGs, as poverty alleviation and promotion of good health no longer received the same priority as before. Besides, the arrival of the COVID-19 pandemic delayed the efforts agreed. On the other hand, the Chinese Government strong political commitment to poverty alleviation and people-cantered policies, as reflected in the Healthy China 2030 plan [6], remains unperturbed. It has succeeded in finally lifting the whole Chinese population above the poverty line by 2020 prolonging the average life expectancy from 35 years in 1949 to 77.8 today. In addition, the elimination of malaria transmission in China was declared by the World Health Organization (WHO) in June 2021 [7]. Against this background, IDP continues to emphasize the publication of papers with a focus on scientific evidence for global poverty reduction targeting SDG indicators relevant to infectious diseases and poverty, advancing technologies improving population health, implementing policy translation of high-quality scientific output and promoting trans-disciplinary communication.

### Promotion of quality-publication thanks to the editorial board members

In order to publish high-quality articles with a wide impact on global health development, the members of the editorial board have been carefully chosen according to expertise on the diseases that affect poor people in poor countries. The IDP editorial board members consists of 58 professional leaders and senior managers from international organizations and disease control programmes from 25 countries, who have actively contributed to organizing thematic series, inviting review articles and formulating scoping review guidelines. These outstanding scientists represent a broad knowledge base, which has been well received by the many young scientists working as section editors.

During its first ten years, the journal has been a movable vanguard producing 36 thematic series categorized into four different types of approaches. The first providing information for the control of infecious diseaes, such as Ebola, malaria and most recently COVID-19; the second consisting of papers on the development of drug efficacy and resistance with regard to malaria, coronavirus and other infections; the third providing a platform for innovative research with trans-disciplinary papers, such as social innovation to transform health care delivery and the economic impact of infectious diseases of poverty, while the fourth focused on scoping reviews covering health system, health policy and disease control strategies. Examples of the latter include urban health, prevention and control of vector-borne diseases, health systems research and other approaches to control and eventually eliminate infectious diseases of poverty.

### Capacity building through training courses and research forums

In order to improve communication among scientists as well develop their capacity of writing scientific papers, reporting on infectious diseases of poverty in particular, IDP organized six medical writing workshops during the last 10 years with more than 300 physical participants in total. Four visual “IDEA Forum—lessons from authors” were offered amid COVID-19 pandemic [8]. The topics covered global response to local poverty-related disease priorities, development of sustainable financing for effective tuberculosis service delivery and more recently relevant study design and mathematical modelling with focus on COVID-19. Through interactions among readers, authors, editors and more than 3,600 participants from various parts of the world, young researchers have learnt how to select research topics and better communicate research results.

## Perspectives for the next decade

Looking forward to the next 10 years, new and additional goals have been formulated after consultancy with senior editorial board members.

First, keeping in line with the SDGs and with WHO's “triple billion” targets is a priority governing IDP’s future strategy and further improve the publication quality. This will be achieved by promoting the “One Health” trans-disciplinary, multisectoral, cross-regional research approach. All stakeholders involved should be coordinated to address issues and scientific evidence with the particular aim of making progress towards the SDG-1, SDG-3 and SDG-6 targets.

Second, further expansion of the journal’s influence at the global level is the next step. It should be realized by publishing articles covering a broader field rather than focusing on a specific question, articles should attempt to cover a collection of activities, e.g., from epidemiological investigation to the policy formulation, or from technique development to field assessment. Encouraging more published information of this kind would encourage disease control programmes to rapidly move new, advanced control technologies into field use. This approach would also accelerate trans-disciplinary research and lead to invigorated presence at international conferences and stronger advocacy as well as stronger marketing of IPD’s thematic series and forum discussions. In addition, shortcomings in the editorial process revealed by SWOT (strengths, weaknesses, opportunities, and threats) analysis will be taken *ad notam*, and we plan to produce cost-effective outcomes in control and elimination of the infectious diseases of poverty.

Third, IDP’s unique features and the continued publication of high-quality articles will be maintained. Submitted manuscripts that fit the IDP scope will be given a fast-forward lane to publication illustrating the need to identify first-rate research and important findings. Identification of potential gaps in our current understanding of research and public health action would be a promoter of scientific evidence for rapid policymaking or policy translation.

A good peer review coupled with additional assistance of directly involved associate editors will not only present guidance with respect to research priority setting or programme decision, but also contribute to the creation of a format that can easily be understood by a wide readership. In order to contribute more to the reduction of disease burdens through the One Heath approach, IDP will in 2022 introduce a new type of article entitled “*Policy Brief”* that will present a focus on design, formulation, implementation, as well as evaluation of control policies that in due course will lead to disease elimination.

It can be concluded that IDP has achieved much in its first 10 years and indeed contributed to improved control and reduction of the infectious diseases of poverty. However, lessons were also learnt on how to elevate impact, sustain and apply research findings in the field, promote quality of articles and thematic series and contribute to better capacity building. In the next decade, IDP will continue to make efforts contributing to achieving those SDGs that coincide with the journal’s original goals with global vision. It will maintain its unique features to further improve scientific impacts and the launch of “*Policy Brief*” that will provide useful recommendations on design, formulation, implementation, and evaluation of policies, which will no doubt lead to rapid translation of research results into routine application.

## Data Availability

Not applicable.

## Abbreviations

IDP: *Infectious Diseases of Poverty*
COVID-19: Coronavirus disease 2019
UHC: Universal Health Coverage
MDGs: Millennium Sustainable Goals
SDGs: Sustainable Development Goals
UN: United Nation
NTDs: Neglected tropical diseases
WHO: World Health Organization
